# A Nuclear Magnetic Resonance (NMR) Platform for Real-Time Metabolic Monitoring of Bioprocesses

**DOI:** 10.3390/molecules25204675

**Published:** 2020-10-13

**Authors:** Ninad Mehendale, Felix Jenne, Chandrakant Joshi, Swati Sharma, Shyam Kumar Masakapalli, Neil MacKinnon

**Affiliations:** 1 Institute of Microstructure Technology, Karlsruhe Institute of Technology, Eggenstein-Leopoldshafen, 76344 Baden-Württemberg, Germany; ninad@somaiya.edu (N.M.); felix.jenne@partner.kit.edu (F.J.); 2 K. J. Somaiya College of Engineering, Somaiya Vidyavihar University, Mumbai, Maharashtra 400077, India; 3 BioX Center, School of Basic Sciences, Indian Institute of Technology Mandi, Kamand, Himachal Pradesh 175075, India; chandrakant.iitmandi@gmail.com (C.J.); shyam@iitmandi.ac.in (S.K.M.); 4 School of Engineering, Indian Institute of Technology Mandi, Kamand, Himachal Pradesh 175075, India; swati@iitmandi.ac.in

**Keywords:** NMR-compatible bioreactor, automated NMR, bioprocess monitoring, microbial bioprocess, waste degradation

## Abstract

We present a Nuclear Magnetic Resonance (NMR) compatible platform for the automated real-time monitoring of biochemical reactions using a flow shuttling configuration. This platform requires a working sample volume of ∼11 mL and it can circulate samples with a flow rate of 28 mL/min, which makes it suitable to be used for real-time monitoring of biochemical reactions. Another advantage of the proposed low-cost platform is the high spectral resolution. As a proof of concept, we acquire ${}^{1}$H NMR spectra of waste orange peel, bioprocessed using *Trichoderma reesei* fungus, and demonstrate the real-time measurement capability of the platform. The measurement is performed over more than 60 h, with a spectrum acquired every 7 min, such that over 510 data points are collected without user intervention. The designed system offers high resolution, automation, low user intervention, and, therefore, time-efficient measurement per sample.

## 1. Introduction

Nuclear Magnetic Resonance (NMR) spectroscopy is an important analytical technique in both material and medical sciences. Based on the quantum mechanical property of spin, NMR is sensitive to the local magnetic/chemical environment with atomic resolution. NMR interaction energies are quite small and they rely on radio frequencies (RF) as probe radiation. As a result, NMR is non-destructive and non-invasive, making it a perfect tool for real-time investigations of the molecular composition of bioreactions [1,2]. The value of such information is intrinsically high, with the potential to reveal opportunities for reaction optimization towards the desired end result, for example, the production of an industrially relevant product in the case of bio-based waste processing [3].

Since the 1990s, there have been many reports using NMR spectroscopy for the purpose of real-time reaction monitoring [4,5,6,7,8,9]. A primary objective has been metabolite measurement of the biological sample located directly within the NMR magnet in order to characterize metabolic function [10,11,12,13,14,15,16,17]. In order to accurately reflect the true metabolism and not a stressed state as a result of the measurement system, the capability to maintain a sample in its natural state is essential, with additional value possible when including the potential for (bio)chemical treatment. Therefore, most systems implement a perfusion system [8,16,18,19] and, in the case of cell cultures, a scaffold for cell immobilization to ensure optimal product and waste diffusion properties [9,10,12,13,14,20]. It is clear that these systems work well; however, several challenges remain:

- the most sensitive and ubiquitous NMR nucleus (${}^{1}$H) remains challenging. Because water is the solvent, efficient solvent suppression techniques must be used, since deuterated solvents at high concentrations often interfere with the biology and can be costly [21]. *In situ* samples are heterogeneous and magnetic susceptibility differences at interfaces prohibit the measurement of high-resolution spectra. This is circumvented by avoiding ${}^{1}$H and instead, measuring nuclei with larger chemical shift dispersion (e.g., ${}^{13}$C and ${}^{31}$P), at the cost of decreased sensitivity (unless hyperpolarization is implemented [14]) and metabolite coverage [22];
- intra- and extra-cellular compartments are simultaneously measured when cultures are loaded into the NMR detector. Situations in which it is important to distinguish signals that arise separately from the two compartments require additional considerations [23];
- absolute quantification requires the addition of a concentration standard. This can be added directly to the perfusion medium, thus potentially interfering with the sample [21] and adding to the cost, or inserted into the sample container in a sealed capillary, further decreasing the spectral resolution.

An alternative to directly monitoring the bioprocess within the NMR sample container is to transport sample aliquots from a bioreactor outside of the NMR system to the NMR detector. When set up appropriately, this configuration enables the measurement of the culture medium exclusively. Therefore, high-resolution spectra are achievable of molecules being consumed by, and excreted from, the biological sample in the bioreactor. This approach has been successfully implemented to monitor chemical reactions [24,25,26,27,28,29,30], including the use of low-field benchtop NMR spectrometers for both chemical [31,32] and biological applications [33,34]. Importantly, the interface to the NMR spectrometer is simplified in comparison with the *in situ* operation mode: only tubing and pumps are required for sample shuttling from a suitable external bioreactor station; in effect, the biological culturing becomes a module to be coupled to the NMR measurement system. This operating mode is an attractive alternative in cases where the monitoring molecular content of culture medium is required.

In this contribution, we demonstrate real-time monitoring of a bioreaction by NMR spectroscopy while using a flow shuttling system to deliver the sample to and from a bioreactor placed outside of the NMR magnet. Our platform enables automated NMR data acquisition by including an interface, between the shuttling system and the NMR spectrometer, to synchronize sample delivery and data acquisition. The flow cell is a concentric glass tube design, allowing for an internal standard solution to be separated in the outer volume from the sample flowing through the central capillary. This platform has the following features:

- High-field compatibility: the flow cell uses a standard 5 mm NMR sample tube as the outer sample container, making it compatible with standard high magnetic field NMR probes.
- High-resolution ${}^{1}$H NMR: The flow cell permits the use of a D${}_{2}$O solution containing an internal standard, thus standard field locking is possible. Although the effective filling factor of the sample solution is reduced in this design, solvent suppression is less complicated, and radiation damping effects can be reduced. The geometry of the flow cell is such that high static magnetic field homogeneity is maintained in the sample region, with minor corrections resolved using the NMR shim system.
- Improved measurement time / sample: because only the liquid sample is transported while the sample container remains within the magnetic field, a system re-calibration (tuning/matching, shimming) for each sample is not required.
- Less user input required: once the platform has been set up and the first NMR experiment is started, there is very little user intervention required.

**Figure 1:**
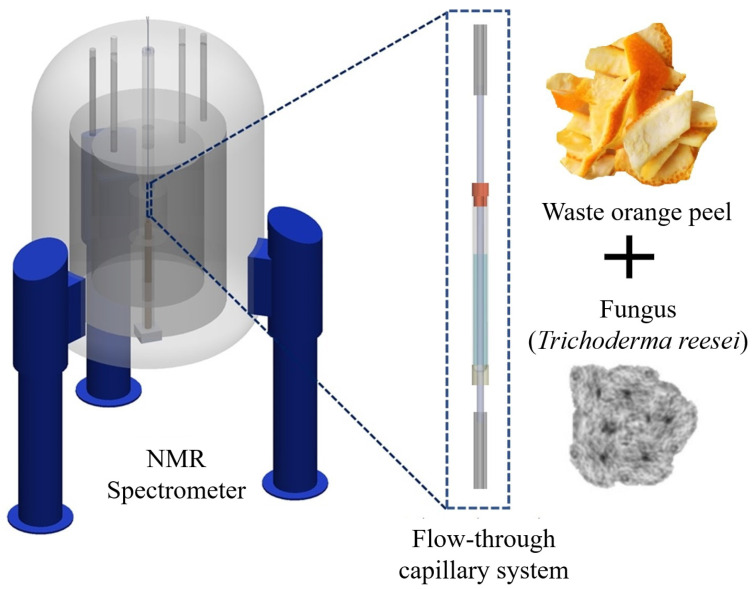
Conceptual diagram of the real-time Nuclear Magnetic Resonance (NMR) measurement system using a flow-through capillary arrangement. The processing of waste orange peel, with the help of (*T. reesei*) in minimal medium, was the bio-reaction to demonstrate the system functionality.

The system operation was verified by a proof-of-concept bio-reaction that was inspired by the current global challenge of the conversion of cellulosic waste into useful chemicals such as lactic acid by microbial bioprocessing. Orange peel was used as the waste material for this purpose. The bioreactor was loaded with waste orange peel and then inoculated with *Trichoderma (T.) reesei* QM6a (Figure 1), a fungus that is known to release significant quantities of cellulases, enzymes that are capable of degrading cellulose (poly-glucose) to glucose and small saccharides [35,36]. The culture medium was then monitored under automation for over 60 h, with one ${}^{1}$H NMR spectrum acquired every 7 min. (including time for sample transport and data acquisition) for more than 510 time-point measurements. The platform is ideal for bioprocessing applications, in which conversion to a value-added product is the objective, since the intermediate and final product yields can be monitored in real-time.

## 2. Materials and Methods

### 2.1. Equipment and Chemicals

Cobalt(II) chloride, anhydrous, 97%, Manganese (II) sulphate tetrahydrate, 99% and Zinc sulphate heptahydrate, ACS, 99.0–103.0% were obtained from Alfa Aesar, Thermo Fisher, Germany. Ferrous sulphate, heptahydrate, Ammonium sulphate ≥99%, and Potassium sulphate ≥99% were acquired from Acros Organics, VWR, Germany. Calcium chloride, ≥98%, and Magnesium sulphate heptahydrate, ≥99%, were obtained from Carl Roth, Germany. 3-(trimethylsilyl)propionic-2,2,3,3-d${}_{4}$ acid sodium salt (TSP) was obtained from Sigma Aldrich. *T. reesei* QM6a was obtained from DSMZ-German Collection of Microorganisms and Cell Cultures (DSM No.:768) and it was maintained on potato dextrose agar. The silicon tubing (Tygon GZ-96460-28) used for the peristaltic pump was obtained locally. All of the NMR experiments were performed on a Bruker AVANCE III 500 MHz wide-bore NMR spectrometer (Bruker BioSpin, Rheinstetten, Germany). The personal reaction station was acquired from J-KEM Scientific.

### 2.2. Sample Insert Design

**Figure 2:**
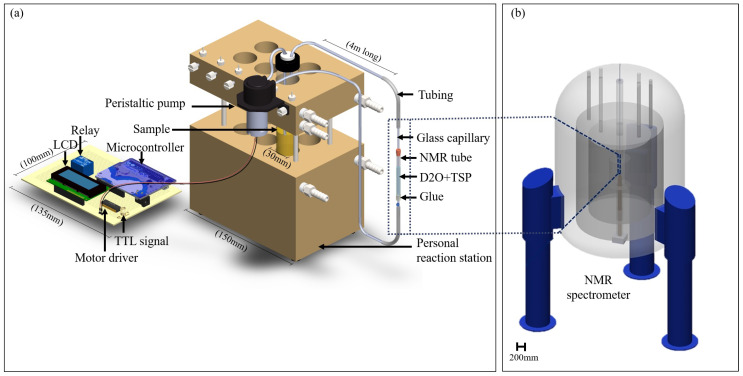
(**a**) Experimental setup. The bioreaction is performed using the personal reaction station (PRS). A peristaltic pump is mounted on the PRS to flow the sample periodically through the capillary-based flow cell (inside the NMR magnet) for NMR measurement and subsequently return it to the bioreactor. The peristaltic pump is controlled by a microcontroller using a relay and a motor driver, with a liquid crystal display (LCD) used to display the time to the user. A TTL (transistor-transistor logic)signal is sent to the NMR spectrometer after the sample has been transferred to the NMR detector to initiate measurement. Note: tubing length not to scale. (**b**) NMR spectrometer with flow-through capillary system in the center.

The NMR insert was designed to be compatible with the Bruker Micro5 microimaging probe (Figure 2).The insert consisted of a glass capillary (2.2 mm o.d. and 1.5 mm i.d.) as the main channel carrying the sample. This capillary was inserted concentrically into a 5 mm diameter standard NMR sample tube that had been cut, so as to be open on both ends. The top and the bottom of the insert were sealed using a three-dimensional (3D) printed cap, which had 2.2 mm holes to accommodate the internal capillary, and glue (Figure 2).The capillary was connected to tubing (Tygon tubing with inner diameter of 1.5 mm) from the bottom, for the sample to be inserted. Another tube was connected to the top of the capillary, which served as an outlet. A solution of TSP in D${}_{2}$O (450 μL, 5 mM TSP) was added in the volume between the central capillary and inner wall of the 5 mm tube for the purpose of shimming and calibration.

### 2.3. Personal Reaction Station

The personal reaction station (PRS), as shown in Figure 2a, was used to maintain the bio-reaction. It provided precise temperature control via a PID controller. In cases where reaction stirring is important, the PRS features magnetic stirring for each reaction tube; for our system, active stirring was not used to avoid destruction of the fungal mycelia [37]. The pumping action ensures a gentle mixing of the medium.

### 2.4. Sample Transport

A low-cost peristaltic pump, along with the in-house built microcontroller-based driving circuitry, was used to transport the sample. The pump was mounted on the PRS to keep the pump stationary during pumping, as shown in Figure 2a. An Arduino microcontroller was used to program the pumping of the sample through the NMR system and to synchronize the measurements (control code available in the Supplementary Material). The controller managed the sample transport direction and the flow rate. Although this configuration allows one to implement bi-directional flow, the system was used for uni-directional sample transport.

The driver circuit consisted of an L293 Driver for driving the DC motor of the peristaltic pump. The driver allowed for us to control the speed and direction of the DC motor. An IC 7805 regulator was used to provide the standard 5 V, 1 amp supply to the entire circuit. The circuit powered by a 12 V battery, which was continuously charged with DC power supply (circuit diagram available in the Supplementary Information). The battery was necessary because of the large current requirements due to periodically turning the pump on and off.

After initializing the reaction in the PRS, the resulting solution was passed through the system by the peristaltic pump into the NMR measurement zone. NMR data acquisition was triggered using a TTL (transistor-transistor logic) signal that is generated by the controller so that sample transport and measurement were synchronized.

### 2.5. Sample Preparation

*T. reesei* fungus was taken out of a stock solution stored at −80 °C. The fungus was revived by adding liquid broth in a 15 mL Falcon tube and then incubating it at 28 °C with shaking at 180 RPM.

A minimal medium component was prepared by mixing the following salts and minerals into DI water. One liter of stock solution was prepared, and then 20 mL of minimal media was used per experiment. Following are the chemicals added per liter of DI water: (1) 7.6 g of (NH_4_)_2_SO_4_, (2) 15 g of KH_2_PO_4_, (3) 591 mg of MgSO_4_, (4) 602 mg of CaCl_2_·2H_2_O, (5) 5 mg of FeSO_4_·7H_2_O, (6) 1.6 mg of MnSO_4_·7H_2_O, (7) 1.4 mg of ZnSO_4_·7H_2_O, and (8) 3.7 mg of CoCl_2_ [38].

**Figure 3:**
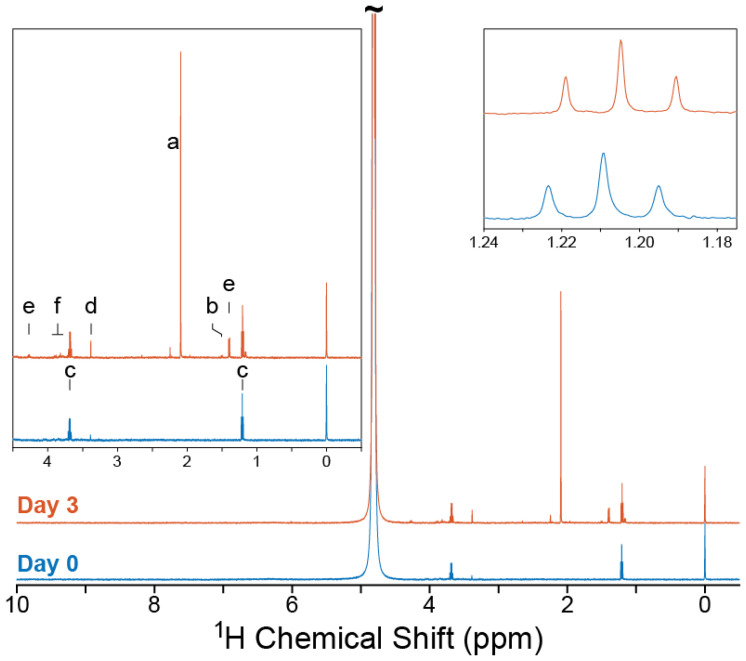
${}^{1}$H NMR spectra obtained with real-time NMR platform. The initial spectrum (Day 0) shows the response obtained before the addition of *T. reesei* to the dried orange peel waste. The final spectrum (Day 3) shows the response obtained on day 3 after the addition of fungus. Inset, left: zoom of the spectral region between −0.5–4.5 ppm, where signals were observed to evolve during the experiment. Inset, right: zoom of the ethanol triplet signal. Abbreviations: (a)—acetate; (b)—alanine; (c)—ethanol; (d)—glycine; (e)—lactate; and, (f)—saccharide.

Waste orange peels were cut to ∼ 5 mm rectangular size, such that they could easily fit inside the PRS tubes. A total of 15 g of waste orange peel was placed inside the PRS glass tube. The tube was then filled with the prepared minimal media. The ${}^{1}$H NMR spectrum was obtained on this sample (Day 0) just after adding the fungus, as shown in Figure 3. A total volume of 31 mL was used during the experiment: 20 mL in the reaction tube, approximately 10.6 mL in the transport lines, and 17.6 μL was used in the NMR detection volume.

### 2.6. Experimental Setup

**Figure 4:**
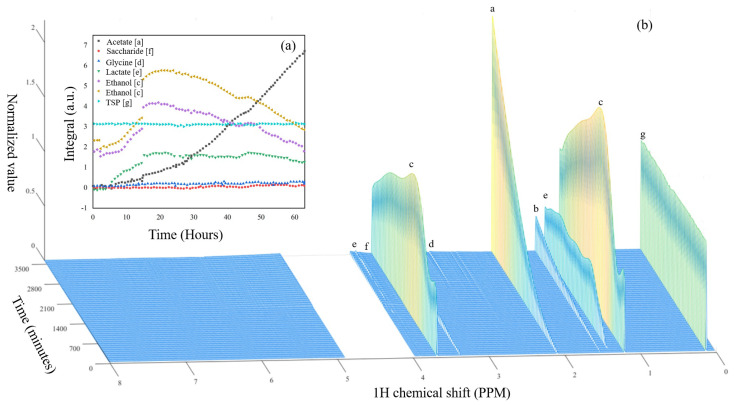
(**a**) Area under the curve for significant peaks seen at the output over three days of continuous real-time NMR monitoring of the bio-reaction of orange peel fed to *T. reesei*. Note: only 80 data points are plotted for clarity. (**b**) Real-time acquired spectra of the 516 runs plotted in 3D with water peak and stationary peaks (except 3-(trimethylsilyl)propionic-2,2,3,3-d${}_{4}$ acid sodium salt (TSP)) removed. Note: only 52 in 516 runs are plotted for better visualization. X-axis is chemical shift (ppm) axis, y axis is time point and z axis is normalized value with respect to TSP. Abbreviations: (a)—acetate; (b)—alanine; (c)—ethanol; (d)—glycine; (e)—lactate; and, (f)—saccharide, (g)—TSP.

The temperature inside the personal reaction station is controllable and it was set to 28 °C, the optimal temperature for the fungal growth. The medium of the bio-reaction was flowed through the system (i.e., from the reaction chamber to the capillary placed inside NMR spectrometer and back to the reaction chamber) for 47 s, which, at a flow rate of 28 mL/min., corresponds to ∼22 mL of solution transported (which was sufficiently larger than the required volume of 10.6 mL of the transport system), before the peristaltic pump was automatically turned off. After a relaxation time of 50 s, the microcontroller sent a TTL signal to the NMR spectrometer in order to begin the data acquisition. Each ${}^{1}$H NMR spectrum contained 128 scans using a 1 s relaxation delay, each containing 32 K data points over a spectral width of 12 ppm, resulting in a total acquisition time of 339 s. The resulting signal was multiplied by an exponential function equivalent to a 0.3 Hz line broadening prior to Fourier transformation. After NMR data acquisition, the microcontroller automatically transported the sample back to the bio-reaction, i.e., the transport lines were emptied. Each repetition of flow, relaxation, and acquisition took approximately 7 min.; hence, every 7 min., a new spectrum of the solution was acquired. In total, 516 NMR experiments were obtained within three days of the experiment. The stacked images are shown in Figure 4. Figure 3 depicts the final resultant scan (Day 3) (orange colored).

## 3. Results and Discussion

The Day 0 ${}^{1}$H NMR measurement, using the automated flow system, is given in Figure 3 (in blue color). From this spectrum, it is noted that high spectral resolution is maintained, as would be expected of a standard liquid state NMR experiment. Additionally, the TSP reference signal at 0 ppm can easily be used for integral normalization and even quantification of the metabolites under appropriate quantitative NMR conditions. The water signal (4.8 ppm) has acceptable line width and minimal baseline distortion, since the flow cell is partitioned into D_2_O and H_2_O volumes. This enables the quantification of signals that would typically not be resolvable when using pure water as the solvent due to the reduction of radiation damping [39]. Finally, residual ethanol was detected in the Day 0 sample (1.20, 3.68 ppm), most likely originating from the sterilization procedure of the PRS and flow lines.

A comparison of Day 0 and Day 3 spectra (Figure 3) reveals the appearance of several new metabolites during the course of the bioreaction, and preliminary assignments are given in Table 1. The kinetic data for selected signals (molecules) are plotted in Figure 4 (spectra plotted in Figure 4b). From this experiment, alanine, acetate, glycine, and lactate could be quantified, with some spectral hints of saccharide signals (potentially fructose and/or glucose as the experiment was performed on fruit waste) appearing near the end of the experiment [40]. Ethanol was also observed to increase in concentration over the first 20 h of the experiment before constantly decreasing to the final time point.

It is notable that the 516 spectra were collected with minimal user intervention. The experiment was interrupted at two points, as reflected in the kinetic data (Figure 4a). At approximately hour 15 and 42, the NMR instrument required a slight adjustment of the shim system in order to correct minor drifts in the magnetic field as is common for long-term experiments. In principle, this intervention can be completely avoided when the NMR lock system is used together with the auto-shim routine, which, in this experiment, could not be used, as our NMR detector was not tunable to deuterium. Nevertheless, with the density of data points, fitting to the kinetic data would still be possible.

The main focus of this work was to verify the suitability of our designed system for long-term, real-time monitoring of an active biological system. The ability to obtain real-time metabolic information on active, non-stressed systems is attractive for several reasons. Given that metabolic function is often different in the diseased or stressed state [42], metabolite profiles can be used to identify dysfunctional states [19] or investigate potential therapeutics [9,17]. The real-time monitoring of metabolite levels could also be exploited for metabolic engineering applications [43], for example, targeting the enhanced production of a value-added product generated from a bio-waste.

It was outside the scope of this work to investigate in detail the *T. reesei* decomposition of the orange peel waste, chosen as a proof-of-concept for our system. It has been demonstrated that significant reducing sugar concentrations (∼50 mM) can be produced by decomposition of whole orange peel at pH 5 and 328 K within 24 h while using *T. reesei* cellulases complex (the enzymes having higher activity at elevated temperature when compared to an ideal fungal culturing temperature of 298-303 K) [40]. Our system also detected industrial products (ethanol, lactate, and acetate) in real time, supporting the fermentation capabilities of *T. reesei* [44]. The reaction conditions that were used in this work were optimal for *T.reesei* cultures and, hence, differed significantly in pH and operating temperature; however, the PRS and NMR flow system could easily be adapted to a variety of operating conditions that are optimized for a particular bioreaction.

We have demonstrated that our real-time NMR monitoring system can successfully operate for more than 60 h without user intervention, with the end of the experiment only defined by the need to hand the NMR instrument over to other users for their experiments. The system has several advantages: since only the sample is transported while the flow cell is stationary, re-adjusting the NMR instrument is not necessary (as would be if, for example, an NMR sample tube exchange system was used, as in [2]); the sample is in a closed loop, therefore avoiding possible contamination; the lock solvent (D_2_O) and chemical shift reference (TSP) are physically separated from the sample and, thus, cannot interfere with the reaction; given the reduced volume fraction of the sample occupying the NMR detection volume, radiation damping is reduced and ${}^{1}$H NMR signals near the water signal can be resolved. There are also features to be improved: the transport lines are currently not insulated and, thus, temperature variation is possible (in our example this is not critical since *T. reesei* is a mesophile with optimal growth conditions 25–30 °C); the transfer lines were relatively long, which, if shortened, could significantly reduce the sample transfer times; our system could not take advantage of the standard NMR lock and auto-shim functions (our NMR instrument limitation, not a limitation of the bioreactor flow system), and thus manual intervention was necessary to correct the magnetic field homogeneity; with the lock enabled, solvent suppression could be additionally used in order to suppress the residual water signal and further enhance sensitivity; given the large metabolite concentration range that was observed in such systems, it would be useful to implement the recently reported AGAIN-NMR method [45].

## 4. Conclusions

A real-time NMR monitoring system was designed to be compatible with a variety of bioreactor configurations, thus achieving a degree of flexibility enabling one to monitor different biochemical reactions. We have demonstrated the real-time measurement capability of the platform by acquiring ${}^{1}$H spectra with high spectral and time resolution of a microbial bioprocess, i.e., orange peel fed to *T. reesei*. Real-time monitoring was accomplished over more than 60 h in this demonstration. An automated interface between the sample shuttling and NMR spectroscopic measurement makes the system user independent after the setup and, thus, the accuracy of the result is high and chances of contamination are very low. This platform enables the possibility of further real-time studies, for example, exploiting the real-time data for the enrichment of a particular product or to monitor the real-time response after application of a stress to the bioreaction.

## Figures and Tables

**Table 1:** The peaks from the ${}^{1}$H NMR spectrum that were acquired during the last run. In ‘s’ stands for a singlet, ‘d’ a doublet, ‘dd’ a doublet of doublets, ‘t’ a triplet, ‘q” a quartet, and ’m’ a multiplet under mutiplicity column. For the multiplets, the peak is equal to the center. Database comparison to the HMDB [41].

Metabolite	Measured (ppm)	Multiplicity	Database (ppm)
Alanine (b)	1.50	d	1.50
Acetate (a)	2.09	s	2.04
Ethanol (c)	1.20	t	1.17
Ethanol (c)	3.68	q	3.65
Glycine (d)	3.38	s	3.54
Saccharide (f)	3.9	m	-
Lactate (e)	1.39	d	1.32
Lactate (e)	4.27	q	4.1

## Supplementary Materials

The code to control the Arduino and circuit diagram is available in the supporting information.
