# Supplementary material for: A Nuclear Magnetic Resonance (NMR) Platform for Real-Time Metabolic Monitoring of Bioprocesses
Source: Molecules. 2020 Oct 13;25(20):4675. doi: 10.3390/molecules25204675 (PMC7587382; doi:10.3390/molecules25204675)

A nuclear magnetic resonance platform for real-time metabolic monitoring of bioprocesses

Ninad Mehendale[1,2], Felix Jenne[1], Chandrakant Joshi[3], Swati Sharma[4], Shyam KumarMasakapalli[3], Neil MacKinnon[1]*

1 Institute of Microstructure Technology, Karlsruhe Institute of Technology, Karlsruhe,Baden-W¨urttemberg, Germany

2 K. J. Somaiya College of Engineering, Somaiya Vidyavihar University, Mumbai,Maharashtra, India 400077

3 BioX Center, School of Basic Sciences, Indian Institute of Technology Mandi,Kamand, Himachal Pradesh, India 175075

4 School of Engineering, Indian Institute of Technology Mandi, Kamand, HimachalPradesh, India 175075

S1.1 MATLAB code for analysis of NMR data: This section provides a detailed MATLAB code that was used to transfer the raw data into a magnitude spectrum between 0 to 12 ppm. The code also includes automatic phase correction and removal of water peak

```
% clear earlier results

clc

close all

clear all

% %% read first time serise (120 experiments)

% fA = fopen(['D:\Users\xv5675\Documents\Data\Transffered\March19\11\211\ser'],'r','n');

% A = fread(fA, 'int32');

% A = reshape(A, [2 length(A)/2]);

% fid1 = complex (A(1,:)',A(2,:)');

% fclose(fA);

% %% read Second time serise

% fA = fopen(['D:\Users\xv5675\Documents\Data\Transffered\March19\11\213\ser'],'r','n');

% A = fread(fA, 'int32');

% A = reshape(A, [2 length(A)/2]);

% fid2 = complex (A(1,:)',A(2,:)');

% fclose(fA);

%

% %% read Third time serise

% fA = fopen(['D:\Users\xv5675\Documents\Data\Transffered\March19\11\214\ser'],'r','n');

% A = fread(fA, 'int32');

% A = reshape(A, [2 length(A)/2]);
```

```
% fid3 = complex (A(1,:)',A(2,:)');
% fclose(fA);
%
% %% read Fourth time serise
% fA = fopen(['D:\Users\xv5675\Documents\Data\Transffered\March19\11\216\ser'],'r','n');
% A = fread(fA, 'int32');
% A = reshape(A, [2 length(A)/2]);
% fid4 = complex (A(1,:)',A(2,:)');
% fclose(fA);
%
% %% read Fifth time serise
% fA = fopen(['D:\Users\xv5675\Documents\Data\Transffered\March19\11\217\ser'],'r','n');
% A = fread(fA, 'int32');
% A = reshape(A, [2 length(A)/2]);
% fid5 = complex (A(1,:)',A(2,:)');
% fclose(fA);
%
% %% Concanitate all time serise togather
% fid=[fid1; fid2; fid3;fid4;fid5];
% % First 320 scans has useful information 120+120+80
% fid_re=fid(1:5242880);
% % Scan 360 onwards has useful information
% fid_rem=fid(5898240:9830400);
% % concate 1 to 320 and then 360 onwards togather
% fid=[fid_re;fid_rem];
% % Initial data points to be neglected for proper furior transform
 fid_init = 68;
% % plot over lapping serise
% figure(1);
% hold off;
% %Decleare X co-ordinates
```

```
 ppm = linspace( -4.037824838389469,15.95674991607666,65536)-1.11;
% % define blank matric to hold magnitude spectrum
MagSpecold=zeros(65536,1);
% % Initialize variable named A2
A2=0;
% % initialize counter
counter=1;
% %To detrmine FID has a valid data for all 516 scans.
valueofFIDs=zeros(516,1);
% %select Valid data serise fid will have all real-time data of all 516 scans
% %over 3 days.
% fid=fid(1:8454144);
% for each individual scan from 0 to 515
load('data.mat');
for i = 515:515
    % tahe 1 fid at a time neglecting initial few vvalues for proper furiou
    % transform
    B = fid((fid_init:16384)+i*16384);
    B = [B; zeros(65536-length(B),1)];
    % Convert signal to fourior domain
    SP = fftshift(fft(B));
    %%%optional step- remove water peak Manually observed and supressed
    %SP(8037:8527)=0;
    %% Phase correction loop
 % exctract real part
    R=real(SP);
 % exctract imaginary part
 I=imag(SP);
 %% Brute-force method with controllable resolution
 for phi=0:0.1:0*2*pi
     % calculate amgnitude at different phase shift
```

```
 MagSpec=abs(R*cos(phi)-I*sin(phi));
 % Determine range and TSP peak magbnitute to finding maxima in that
 % perticular range
 magTSP=real(MagSpec(3794:4610));
 %%%Maxima peak TSP to be determenied for normalization
 %[code to be written]

 %maximum magnitude is preserved
 MagSpecold=max(MagSpec,MagSpecold);
 %%increament counter
 counter=counter+1;
 %plot(real(B));
 %[pks,locs,widths,proms] = findpeaks(MagSpec,ppm);
 %plot ppm vs Magnitude spectrum
 plot(ppm,MagSpec);
 %Optional hold on
 hold on

 end
 %% to determine valide fid magnitude of real part of spectrum was computed
VV=double(abs(real(MagSpec)));
 %% mean of magnitude of real part of spectrum was computed
valueofFIDs(i+1)=mean(VV);
%Copy of maximum magnitude spectrum is stored
A4=MagSpecold;
%plot now with in loop
drawnow
% store All A4 values in concanitated plot
A2=[A2 A4'];
%     plot(ppm,-real(SP));
%     hold on;
```

end

S1.2 Arduino Code: The Arduino code provided is used to set TTL signals after a particular time duration. Also, the same code is used to declare the delays between consecutive TTL signals. These TTL signals are sent periodically to the NMR spectroscope

```
int x = 0;

int time1 = 0;

#include <LiquidCrystal.h>

const int rs = 7, en = 6, d4 = 11, d5 = 10, d6 = 9, d7 = 8;

LiquidCrystal lcd(rs, en, d4, d5, d6, d7);

void setup() {

  pinMode(5, OUTPUT);

  pinMode(4, OUTPUT);

  pinMode(13, OUTPUT);

  lcd.begin(16, 2);

  lcd.print(" BioPEC IMT KIT ");

  delay(2000);

  lcd.setCursor(0, 1);

  lcd.print("Time: ");

  digitalWrite(13, LOW);


}


void loop() {


  time1 = millis() / 1000;

  lcd.setCursor(6, 1);

  lcd.print(time1);

  x = time1 % 3;

  if(x==0)

  {

  digitalWrite(5, HIGH);

  digitalWrite(6, LOW);
```

```
  lcd.setCursor(0, 0);
  lcd.print(" Pumping liquid ");
  delay(150000);
  digitalWrite(5, LOW);
  digitalWrite(6, LOW);
  delay(10000);

  digitalWrite(13, HIGH);
  lcd.setCursor(0, 0);
  lcd.print("   TTL signal   ");
  delay(337000);
  digitalWrite(13, LOW);
  //delay(500);
  //digitalWrite(13, HIGH);

  }
  else
  {
  digitalWrite(5, LOW);
  digitalWrite(6, LOW);
  digitalWrite(13, LOW);
  lcd.setCursor(0, 0);
  lcd.print(" System Standby ");
  }
}
```

S1.3 Circuit diagram for Arduino-based peristaltic pump driver:

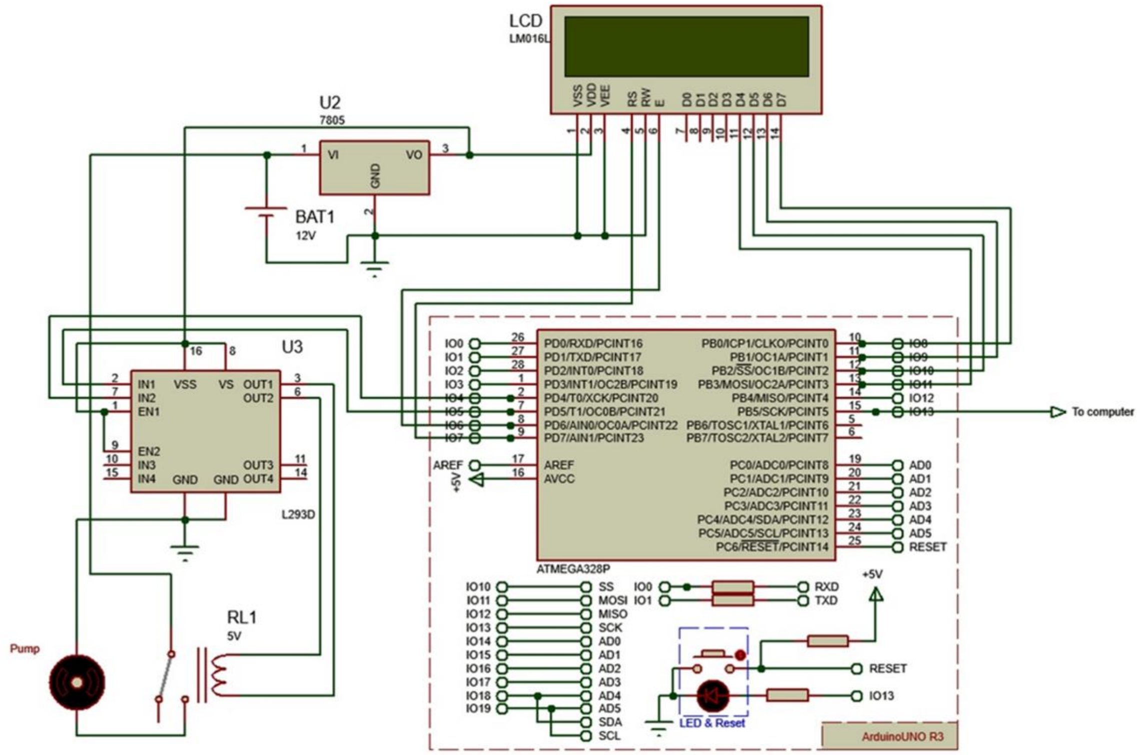

The circuit shows connection details between the microcontroller (Arduino) interfaced with LCD, replay driver, motor. The IC L293D was used as a relay driver and the motor is connected to it directly. The entire circuit is powered by a 12-volt battery and voltage regulator IC 7805. The 16x2 LCD was used to display the timing and the current status of the system. The code for this circuit is given in section S1.2 the main reason to use this circuit was to provide the desired delays in an automated manner.

S2 The 5mm Insert Configuration suitable for any NMR spectrometer

The figure shows a different configuration of insert for NMR spectrometer. This insert was developed using a U-bent capillary of thickness 0.7mm diameter. In this configuration, unlike the flow through system, both input and output tubes were going through the top. The main advantage of such a system was it could have been used with standard NMR spectroscope with regular inserts. Developing a U-bent 0.7mm capillary was challenging also the flow at bent was disturbed hence possible accumulation of the particles. because of these issues, we planned to design the flow through insert system mentioned in the manuscript.

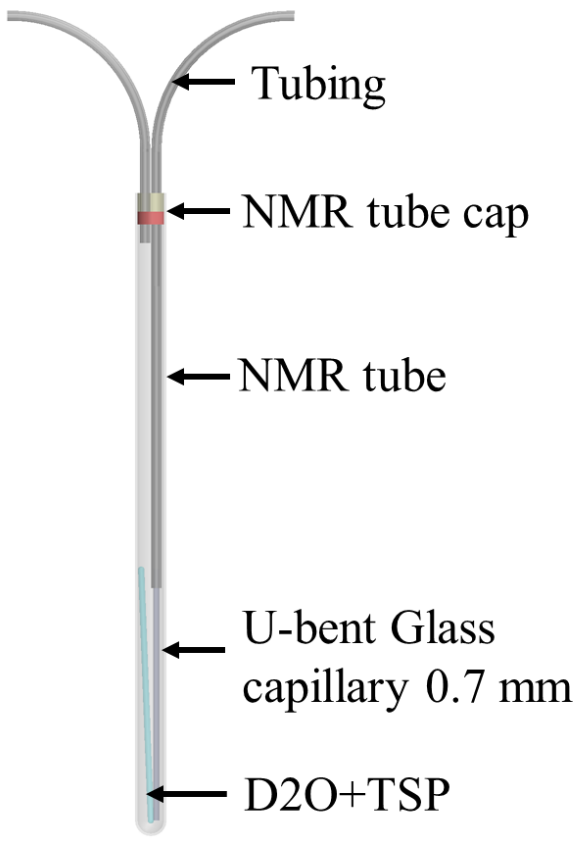

S3 Design of 3D printed filter placed inside bioreaction camber

This filter is used to avoid large wasted particles entering the tubing. The filter had a pore size of 0.5mm. A rectangular grid was printed on Ultimaker-3 which can hold standard filters. This filter was placed just above the solid orange peel waste. The four pillars help to hold the filter at a particular height.

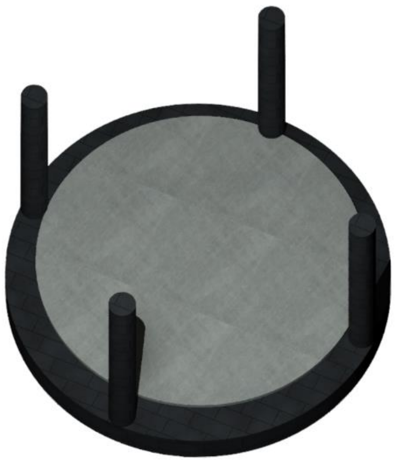

Supplement: Supplementary file 1 [file molecules-25-04675-s001.pdf]
